# Eccentric sombor index of graphs and its role in the structure-property relationship analysis of polycyclic aromatic compounds

**DOI:** 10.1038/s41598-026-36192-z

**Published:** 2026-02-13

**Authors:** Balkishbanu Khaji, Shahistha Hanif, K. Arathi Bhat

**Affiliations:** Manipal Institute of Technology, Manipal Academy of Higher Education, Manipal, India

**Keywords:** Eccentricity, Diameter, Radius, Quality education, Chemistry, Mathematics and computing

## Abstract

This article focuses on the eccentric Sombor index, a variant of the degree-based Sombor index in which vertex degrees are replaced by their eccentricities. Several bounds for this index are established in terms of other known topological indices. Additionally, the practical utility of the eccentric Sombor index is demonstrated through its application in predicting physicochemical properties of polycyclic aromatic compounds. Our analysis reveals that the index shows a strong linear correlation with a coefficient of determination $R^2>0.9$, with key properties such as molecular weight, boiling point, molar refractivity, polarizability, molar volume, and flash point. Furthermore, a comparative study indicates that the eccentric Sombor index offers greater predictive accuracy than the traditional degree-based Sombor index.

## Introduction

In the modern era of explosive growth in nanomaterials, crystalline materials, and drugs, exhaustive lab experiments to explore chemical behaviors burden pharmaceutical scientists. In this context, the computation of various topological indices has emerged as an effective approach for gaining valuable insights into the medicinal properties of a wide range of compounds and drugs^1,2^. Such indices enable the extraction of meaningful chemical and medical information while considerably reducing the need for costly and time-consuming experimental procedures^3,4^.

Chemical graph theory has gained significant popularity among researchers due to its broad applicability in mathematical chemistry. A molecular graph *G*(*V*, *E*) is a simple graph with *n* vertices and *m* edges. In the molecular graph *G*, each vertex $v_i \in V(G)$ represents a non-hydrogen atom and each edge $(v_i, v_j) \in E(G)$ represents a covalent bond connecting those atoms. The molecular graphs of hydrocarbons, which are specifically made up of just carbon and hydrogen atoms, indicate the carbon skeleton of the molecule. The degree $d_u$ of a vertex *u* is the number of its neighboring vertices. The distance between two vertices $v_i$ and $v_j$ in a molecular graph is denoted by $d(v_i,v_j)$, which is the length of the shortest path connecting them. The eccentricity of a vertex $v_i$ is the distance from the farthest vertex $v_j$, that is $\xi (v_i)=max\{d(v_i,v_j): v_i,v_j\in V(G)\}$. The radius and diameter are the two parameters measured from eccentricity defined as $R(G)=min\{\xi (v_i): v_j\in V(G)\}$ and $D(G)=max\{\xi (v_i): v_j\in V(G)\}$, respectively. A graph is self-centered if $D(G)=R(G)$. For more graph-theoretic terms and notations, readers can refer to^5,6^.

The topological index is a numerical descriptor derived from a molecular graph, which helps in characterizing and predicting physicochemical properties of a molecule. Topological indices play a significant role in theoretical chemistry, mainly in quantitative structure-property relationships and quantitative structure-activity relationships analysis^7^. Among the various classes of topological indices, the vertex eccentricity-based topological indices (obtained by replacing the degree of a vertex by its eccentricity) play a crucial role in mathematical chemistry. In 1997, Sharma et al.^8^ introduced the eccentricity-based graphical indices. Over the past few decades, a great deal of study has been conducted in this field. Few eccentricity-based indices are effectively used for mathematical models of a broad spectrum of biological activities^9–11^. Many researchers^12–24^ have studied various eccentricity-based topological indices of molecular graphs and their applications.

The first eccentricity-based index introduced by Sharma et al.^8^ in the year 1997 is the eccentric connectivity index $\xi ^c(G)$ and is defined as

$$\begin{aligned} \xi ^c(G)=\sum \limits _{u \sim v}\xi (u)+\xi (v). \end{aligned}$$

Subsequently, in the year 2012, eccentricity-based first and second Zagreb indices, denoted by $\xi _1^*(G)$ and $\xi _2^*(G)$, were introduced^25^, which are given by

$$\begin{aligned} \xi _1^*(G)=\prod _{u\in V(G)} \xi (u)^2~~~\text { and } ~~\xi _2^*(G)=\prod _{uv\in E(G)} \xi (u) \xi (v). \end{aligned}$$

Another index used effectively by chemists is the forgotten eccentric index $F^{\xi }(G)$^26^, which is defined as

$$\begin{aligned} F^\xi (G)= \sum \limits _{u \sim v} \xi (u)^2+\xi (v)^2. \end{aligned}$$

Recently, in the year 2021^27^, Kulli introduced the eccentric version of the degree-based Sombor index, called the eccentric Sombor index (fourth Sombor index) of a graph *G*, defined as

$$\begin{aligned} SO^{\xi }(G)= \sum \limits _{u \sim v} \sqrt{\xi (u)^2+\xi (v)^2}. \end{aligned}$$

Additional information on the eccentric Sombor index can be found in^28,29^.

In an attempt to explore properties and bounds associated with the eccentric Sombor index, some of the existing results are used, which are stated below.

### Lemma 1

*The Cauchy–Schwarz inequality*^30^*: If *$(a_1,a_2,\dots , a_p)$ *and *$(b_1,b_2,\dots , b_p)$ *are real p-vectors then,*

$$\sum \limits _{i=1}^{p}a_ib_i \le \sum \limits _{i=1}^{p}a_i^2\sum \limits _{i=1}^{p}b_i^2.$$

### Lemma 2

^31^* If *$a_i,b_i\ge 0$ *and *$xb_i \le a_i \le yb_i$ *for *$1 \le i \le n$*, then*

$$\left( \sum \limits _{i=1}^{n}a_i^2\right) \left( \sum \limits _{i=1}^{n}b_i^2\right) \le \frac{(x+y)^2}{4xy}\left( \sum \limits _{i=1}^{n}a_ib_i\right) ^2.$$

*If *$a_i > 0$ *for some *$1 \le i \le n$*, then the equality holds if and only if *$x = y$ *and *$a_i = xb_i$ *for every *$1 \le i \le n$*.*

### Lemma 3

*The Kober’s inequality*^32^*: If *$a_i > 0$ *for *$1 \le i \le j$*, then*

$$\sum \limits _{i=1}^ja_i+j(j-1)\left( \prod \limits _{i=1}^ja_i\right) ^{1/j}\le \left( \sum \limits _{i=1}^{j}\sqrt{a_i}\right) ^2 \le (j-1) \sum \limits _{i=1}^{j}a_i + j \left( \prod \limits _{i=1}^ja_i\right) ^{1/j}.$$

## Motivation for the study

The degree-based Sombor index was initially defined as

1 $$\begin{aligned} SO(G)= \sum \limits _{u \sim v} \sqrt{deg(u)^2+deg(v)^2} \end{aligned}$$

and used by chemists in QSPR analysis of various compounds^33,34^. Soon, this index attracted the interest of mathematicians^35,36^. The Sombor index has gained relevance in network science and has been applied to the modeling of complex dynamical systems in technological, biological, and social contexts^37,38^. To achieve better precision than the previously available ones, the vertex degrees are replaced by their eccentricities in various degree-based topological indices. The eccentric Sombor index is one such index introduced in 2021. The diverse applications of the Sombor index serve as motivation for us to measure its predictive power for the physicochemical properties of polycyclic aromatic compounds.

In the next section, we give the bounds for the eccentric Sombor index in terms of radius, diameter, and also in terms of other eccentricity-based topological indices. Section 4 deals with an application of the eccentric Sombor index in qualitative structure property analysis of polycyclic aromatic compounds. A statistical technique called regression analysis is used to capture the correlation between the dataset containing the physicochemical properties of compounds and the topological indices.

## Bounds for eccentric Sombor index

In this section, some bounds for the eccentric Sombor index in terms of a few significant graph parameters like radius, diameter, are obtained. Further, we established the bounds for the eccentric Sombor index in terms of other graph indices like eccentricity-based Zagreb indices, eccentric connectivity index, forgotten eccentric index, and the multiplicative version of eccentricity-based indices.

### Theorem 1

*Let G* *be a graph with diameter* *D and radius R. Then*

$$SO^{\xi }(G)\le \frac{\sqrt{D^2+R^2}}{D+R}\xi ^c(G)$$

*where* $\xi ^c(G)$ *is the eccentric connectivity index. The equality holds if the graph G* *is self-centered.*

### Proof

Let $m=|E(G)|$. If *G* is self centered, then $D=R$ which implies $SO^{\xi }(G)=\sqrt{2}mD$ and $\xi ^c(G)=2mD(G)$, attaining the equality in the statement of the theorem.

Let $R<D$. It true that $\frac{2DR}{D^2+R^2}\le \frac{2ab}{a^2+b^2}$ for every $a, b \in [R, D]$ with equality if and only if $\{a,b\}=\{r, D\}$. Hence, for every $a, b \in [r, D]$,

$$\frac{(D+R)^2}{D^2+R^2}=1+\frac{2DR}{D^2+R^2}\le 1+\frac{2ab}{a^2+b^2}=\frac{(a+b)^2}{a^2+b^2}.$$

Since for any vertex *u*, it is true that $\xi (u) \in [R, D]$, we can write

$$\begin{aligned} \begin{aligned} \frac{(D+R)}{\sqrt{D^2+R^2}}&\le \frac{\xi (u)+\xi (v)}{\sqrt{\xi (u)^2+\xi (v)^2}}\\ \sqrt{\xi (u)^2+\xi (v)^2}&\le \left( \xi (u)+\xi (v) \right) \frac{\sqrt{D^2+R^2}}{D+R}\\ \sum \limits _{u \sim v}\sqrt{\xi (u)^2+\xi (v)^2}&\le \frac{\sqrt{D^2+R^2}}{D+R}\sum \limits _{u \sim v} \xi (u)+\xi (v)\\ \end{aligned} \end{aligned}$$

Thus, $$SO^{\xi }(G) \le \frac{\sqrt{D^2+R^2}}{D+R} \xi ^c(G).$$ $$\square$$

### Theorem 2

*Let G* *be a graph containing m edges having diameter D* *and radius R. Then*

$$\frac{2\sqrt{ mRD F^\xi (G) }}{D+R} \le \quad SO^{\xi }(G) \quad \le \sqrt{ mF^\xi (G)}$$

*where* $F^\xi (G)$ *is the forgotten eccentric index, and the equality holds if the graph G is self-centered.*

### Proof

From Cauchy-Schwarz inequality 1

$$\begin{aligned} \begin{aligned} \left( \sum \limits _{u \sim v}\sqrt{\xi (u)^2+\xi (v)^2}\right) ^2&\le \sum \limits _{u \sim v}\left( \sqrt{\xi (u)^2+\xi (v)^2}\right) ^2\sum \limits _{u \sim v} 1^2\\ SO^{\xi }(G)^2&\le m \sum \limits _{u \sim v} \xi (u)^2+\xi (v)^2\\ Thus, \quad SO^{\xi }(G)&\le \sqrt{m F^\xi (G) }. \end{aligned} \end{aligned}$$

To prove lower bound, let $a_i=\sqrt{\xi (u)^2+\xi (v)^2}$ and $b_i=1$, then by Lemma 2 we have

$$\begin{aligned} \begin{aligned} \sum \limits _{u \sim v} 1^2 \sum \limits _{u \sim v} \left( \sqrt{\xi (u)^2+\xi (v)^2}\right) ^2&\le \frac{(D+R)^2}{4DR}\left( \sum \limits _{u \sim v}\sqrt{\xi (u)^2+\xi (v)^2}\right) ^2\\ mF^\xi (G) \left( \frac{4DR}{(D+R)^2}\right)&\le SO^{\xi }(G)^2 \\ \frac{2\sqrt{mRD F^\xi (G)}}{D+R}&\le SO^{\xi }(G). \end{aligned} \end{aligned}$$

$$\square$$

### Theorem 3

*Let G* *be a graph having* *m edges with diameter D* *and radius R. Then*

$$SO^{\xi }(G)\ge \frac{ F^{\xi }(G)+2mRD}{\sqrt{2}(D+R)}.$$

*Equality is attained when* *G* *is self-centered.*

### Proof

We have $\sqrt{2}R\le \sqrt{\xi (u)^2+\xi (v)^2} \le \sqrt{2}D$, which implies

$$\begin{aligned} \begin{aligned} \left( \sqrt{\xi (u)^2+\xi (v)^2}-\sqrt{2}R\right) \left( \sqrt{2}D-\sqrt{\xi (u)^2+\xi (v)^2}\right)&\ge 0\\ \sqrt{2}D\sqrt{\xi (u)^2+\xi (v)^2}-(\xi (u)^2+\xi (v)^2)-2RD+\sqrt{2}R\sqrt{\xi (u)^2+\xi (v)^2}&\ge 0\\ \sqrt{2}(D+R)\sqrt{\xi (u)^2+\xi (v)^2}-(\xi (u)^2+\xi (v)^2)-2RD&\ge 0\\ \sqrt{2}(D+R)\sqrt{\xi (u)^2+\xi (v)^2}&\ge (\xi (u)^2+\xi (v)^2)+2RD\\ \sum \limits _{u \sim v}\sqrt{\xi (u)^2+\xi (v)^2}&\ge \frac{1}{\sqrt{2}(D+R)}\sum \limits _{u \sim v} \xi (u)^2+\xi (v)^2+ \sum \limits _{u \sim v} 2RD\\ SO^{\xi }(G)&\ge \frac{ F^\xi (G)+2mRD}{\sqrt{2}(D+R)}. \end{aligned} \end{aligned}$$

$$\square$$

### Theorem 4

*Let* *G* *be a graph with the eccentric connectivity index *$\xi ^c(G)$*. Then*

$$SO^{\xi }(G)\ge \frac{\xi ^c(G)}{\sqrt{2}}.$$

### Proof

By the root mean square and arithmetic mean inequality, $\sqrt{\frac{a^2+b^2}{2}} \ge \frac{a+b}{2}$

On substituting $a=\xi (u)$ and $b=\xi (v)$, we get

$$\begin{aligned} \begin{aligned} \sqrt{\frac{\xi (u)^2+\xi (v)^2}{2}}&\ge \frac{\xi (u)+\xi (v)}{2}\\ \sqrt{2} \sum \limits _{u \sim v}\sqrt{\xi (u)^2+\xi (v)^2}&\ge \sum \limits _{u \sim v} \xi (u)+\xi (v)\\ SO^{\xi }(G)&\ge \frac{\xi ^c(G)}{\sqrt{2}}. \end{aligned} \end{aligned}$$

$$\square$$

The next bound is in terms of the eccentricity version of the first multiplicative hyper Zagreb index, $HM_{1}(G)=\prod \limits _{u\sim v} \left( deg(u)+deg(v)\right) ^2$. The multiplicative version of Zagreb indices was introduced in 2012^39^, and it is proved that $HM_{1}(G)$ is minimal when *G* is a path graph. The eccentricity-based first multiplicative hyper Zagreb index, denoted by $HM^{\xi }_{1}(G)$ is given by

$$HM^{\xi }_{1}(G)=\prod \limits _{u\sim v} \left( \xi (u)+\xi (v)\right) ^2.$$

### Theorem 5

*Let* *G be a graph containing m* *edges with *$F^\xi (G)$*,* $\xi _2^*(G)$ *and* $HM^{\xi }_{1}(G)$ *being the forgotten eccentric index, the second Zagreb eccentricity index and the eccentricity-based first multiplicative hyper Zagreb index, respectively. Then*

$$\sqrt{ F^\xi (G)+m(m-1)\root m \of {HM^{\xi }_{1}(G)-2\xi _2^*(G)}}\le SO^{\xi }(G)\le \sqrt{ F^\xi (G)(m-1)+m\root m \of {HM^{\xi }_{1}(G)-2\xi _2^*(G)}}.$$

### Proof

Substituting $a_i=\xi (u)^2+\xi (v)^2$ in Kober’s inequality, we get

$$\begin{aligned} \begin{aligned} \left( \sum \limits _{u \sim v}\sqrt{\xi (u)^2+\xi (v)^2}\right) ^2\ge&\sum \limits _{u \sim v}{\xi (u)^2+\xi (v)^2} + m(m-1) \left( \prod \limits _{u \sim v}\xi (u)^2+\xi (v)^2\right) ^{1/m}\\ SO^{\xi }(G)^2\ge&F^\xi (G) + m(m-1) \left( \prod \limits _{u \sim v}(\xi (u)+\xi (v))^2-2\xi (u)\xi (v)\right) ^{1/m}\\ =&F^\xi (G) + m(m-1) \left( \prod \limits _{u \sim v}(\xi (u)+\xi (v))^2 - 2 \prod \limits _{u \sim v} \xi (u)\xi (v)\right) ^{1/m}\\ =&F^\xi (G)+m(m-1)\root m \of {HM^{\xi }_{1}(G)-2\xi _2^*(G)}\\ SO^{\xi }(G) \ge&\sqrt{F^\xi (G)+m(m-1)\root m \of {HM^{\xi }_{1}(G)-2\xi _2^*(G)}}.&\end{aligned} \end{aligned}$$

Also,

$$\begin{aligned} \begin{aligned} \left( \sum \limits _{u \sim v}\sqrt{\xi (u)^2+\xi (v)^2}\right) ^2\le&(m-1)\sum \limits _{u \sim v}{\xi (u)^2+\xi (v)^2} + m \left( \prod \limits _{u \sim v}\xi (u)^2+\xi (v)^2\right) ^{1/m}\\ SO^{\xi }(G)^2\le&(m-1) F^\xi (G) + m \root m \of {HM^{\xi }_{1}(G)-2\xi _2^*(G)}\\ SO^{\xi }(G) \le&\sqrt{(m-1)F^\xi (G)+m\root m \of {HM^{\xi }_{1}(G)-2\xi _2^*(G)}}.&\end{aligned} \end{aligned}$$

$$\square$$

## Structure property analysis of polycyclic aromatic hydrocarbons using the eccentric Sombor index

Polycyclic aromatic hydrocarbons are organic compounds composed of two or more fused benzene rings, in which adjacent rings share a pair of carbon atoms, and contain no heteroatoms or substituent groups. Based on the number of fused rings, polycyclic aromatic hydrocarbons are classified into light polycyclic aromatic hydrocarbons, which contain up to four fused rings, and heavy polycyclic aromatic hydrocarbons, which comprise more than four fused rings. In comparison with light PAHs, heavy PAHs exhibit enhanced stability and significantly higher levels of toxicity^40^. The structural arrangement of benzene rings in PAHs gives rise to a wide range of physical, chemical, and toxicological properties. These ring systems can adopt various configurations and may occur either with or without substituent groups. Polycyclic aromatic hydrocarbons vary from semi-volatile to high-boiling compounds. Many researchers have studied the QSPR analysis of polycyclic aromatic hydrocarbons^41–43^.

**Fig. 1:**
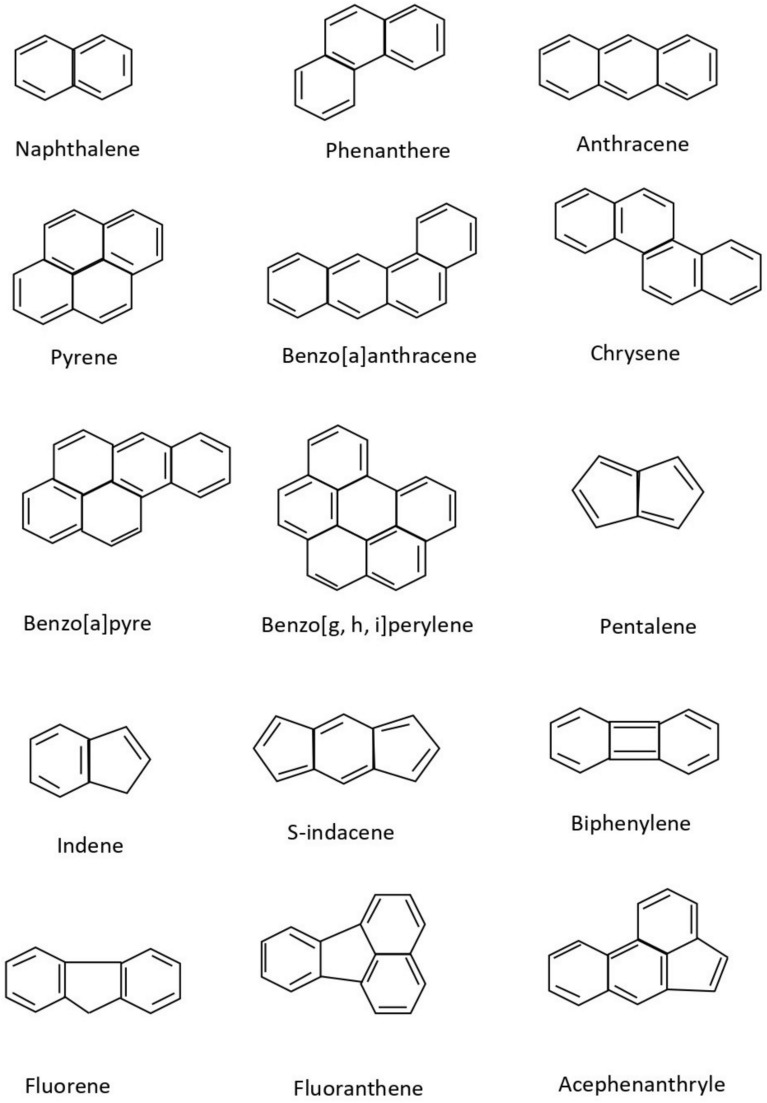

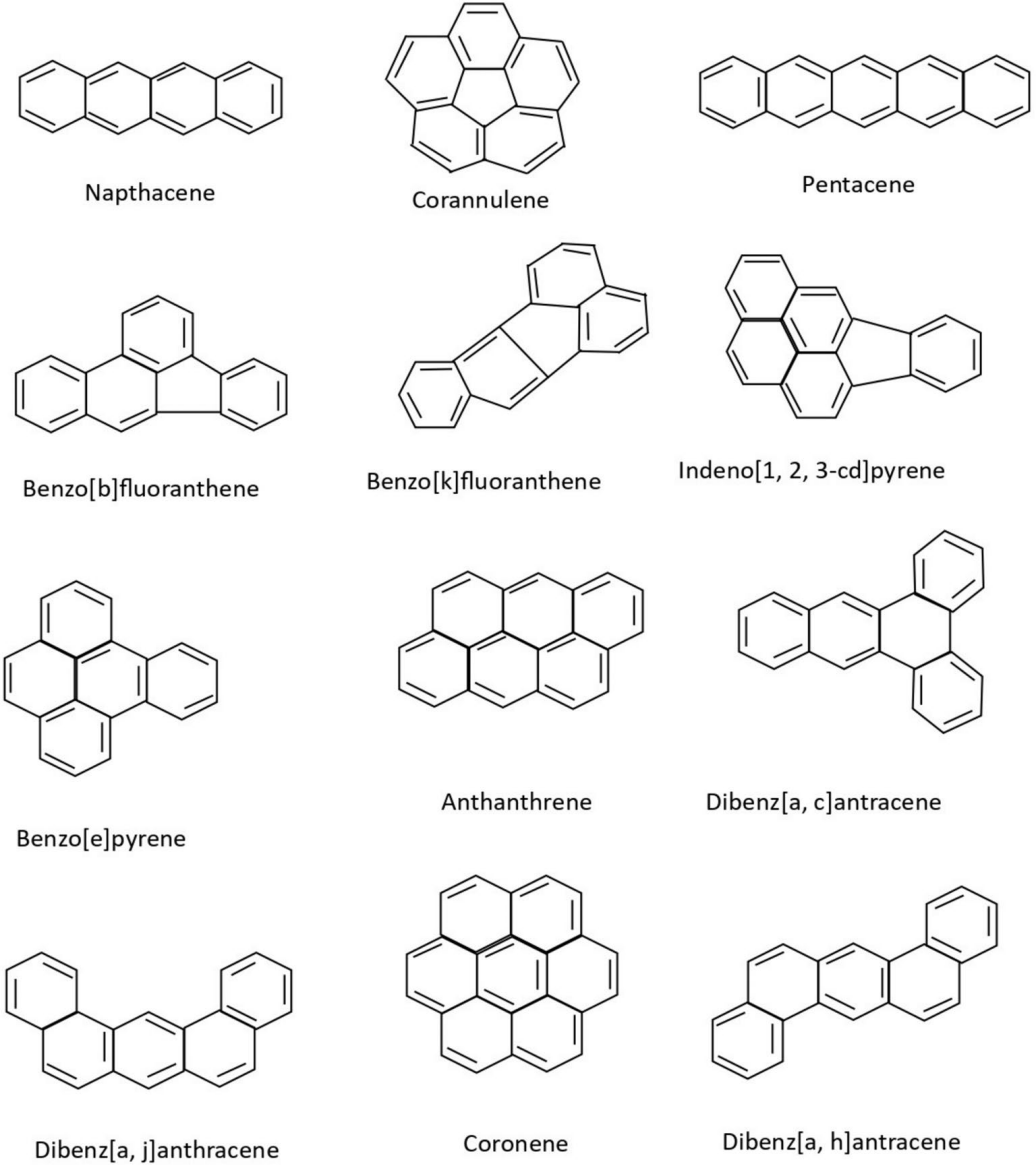

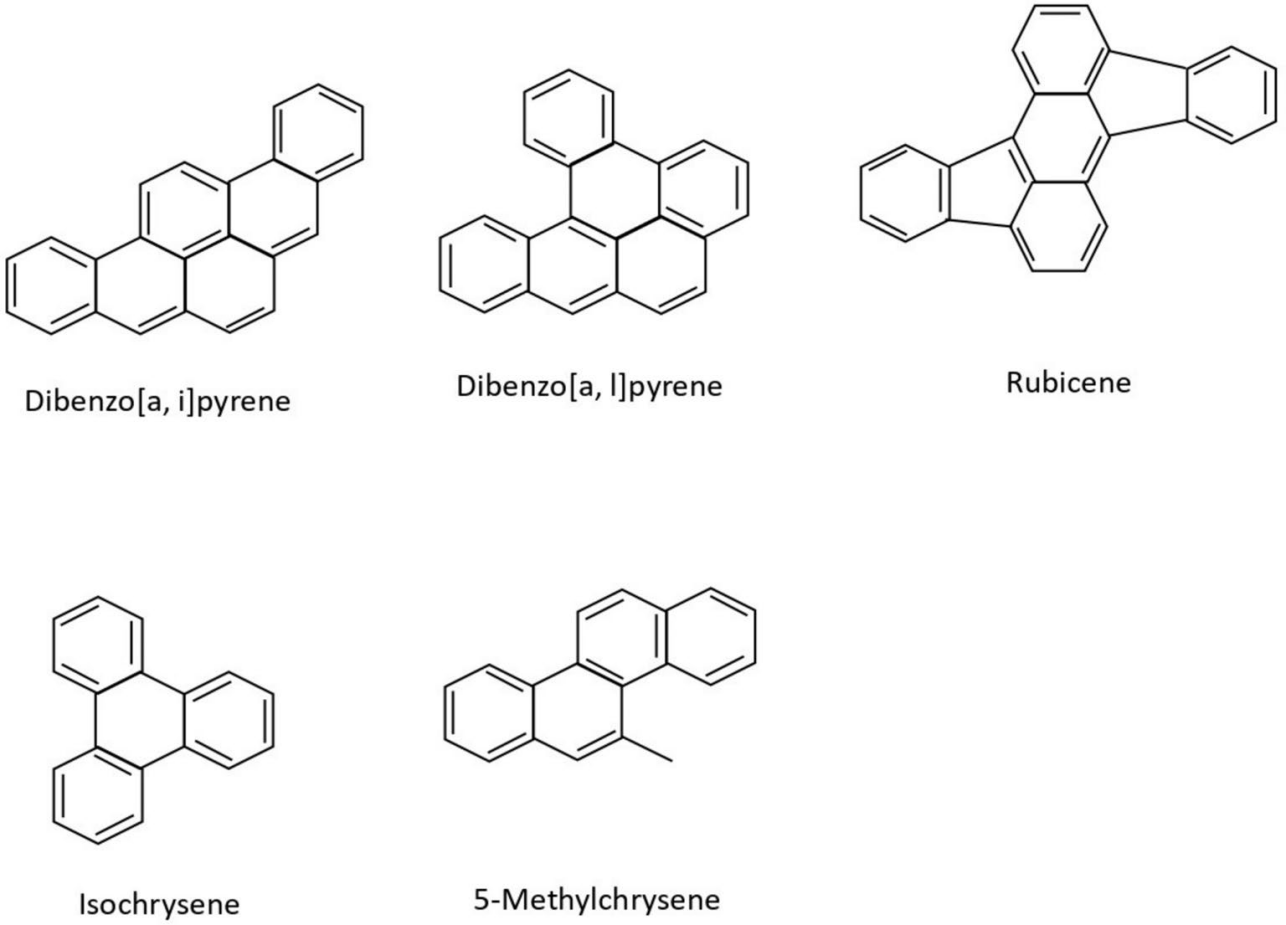
Some polycyclic aromatic compounds.

Although polycyclic aromatic hydrocarbons are not typically synthesised on an industrial scale, several of them are used in commercial applications. Their primary uses include acting as intermediates in the production of photographic materials, thermosetting plastics, pharmaceuticals, agricultural chemicals, and various other products. Polycyclic aromatic hydrocarbons may be present as impurities in carbon black dyes. In particular, naphthalene frequently occurs as an impurity originating from low-quality raw materials used as intermediates in the production of textile dye-dispersing agents and can also be detected in finished textile products. Some of the well-known polycyclic aromatic compounds, having a wide variety of applications, are given in Fig. 1. In this article, a predictive analysis of the eccentric Sombor index among polycyclic aromatic compounds is undertaken.

### Regression models

Regression analysis is a statistical technique widely used in finance, investment studies, and many other fields to examine the relationship between a dependent variable and one or more independent variables. Among the various regression models, linear regression is the most commonly applied to identify the best-fitting straight line that describes the observed data based on a defined mathematical criterion. Typically, linear regression employs the least-squares method, which determines the optimal fit by minimising the sum of squared deviations between the observed values and those predicted by the model. The coefficient of determination is denoted by $R^2$, and it measures how effectively a linear model explains the relationship between variables as depicted in a scatter plot. Specifically, $R^2$ measures the extent to which the model’s predictions correspond to the observed data and ranges from 0 to 1, representing 0% to 100% of the total variation explained by the model.

The regression model is designed to establish and quantify the relationship between eccentric Sombor index with molecular properties, like boiling point BP ($$^{0}C$$), melting point MP ($$^{0}C$$), molecular weight MW(g/mol), molar refractivity MR ($$cm^3$$), flash point FP ($$^{0}C)$$, molar volume MV ($$cm^3$$) and polarizability PO ($$10^{-24}cm^3$$). The empirical data (source: PubChem database) containing the properties of various PAHs used for the analysis along with the calculated eccentric Sombor index is given in Table 1.

**Table 1:** Polycyclic aromatic compounds with their respective physico-chemical properties and calculated eccentric Sombor index.

Chemical compound	MW	MP	BP	MR	PO	MV	FP	$SO^{\xi }(G)$
Napthalene	128.17	81	221.5	44.1	17.5	123.5	78.9	63.9973
Phenanthrene	178.23	100	337.4	61.9	24.6	157.7	146.6	120.6017
Anthracene	178.23	–	337.4	61.9	24.6	157.7	146.6	127.6727
Pyrene	202.25	150	404	72.5	28.7	162	168.8	141.8149
Benzo[a]anthracene	228.3	161	436.7	79.8	31.6	191.8	209.1	201.2868
Chrysene	228.3	255	448	79.8	31.6	191.8	209.1	198.4584
Benzo[a]pyrene	252.3	176	495	90.3	35.8	196.1	228.6	225.3877
Benzo[g,h,i]perylene	276.3	273	501	100.8	40	200.4	247.2	230.9808
Pentalene	102	–	308.9	34.2	13.6	96.1	97.2	37.2506
Indene	116.16	−2	181.6	38	15.1	111.8	58.9	49.6984
S-indacene	152.19	–	–	50	19.8	129.9	–	88.1672
Biphenylene	152.19	115	–	50	19.8	129.9	–	96.4514
Fluorene	166.22	115	293.6	53.8	21.3	148.3	133.1	102.1083
Fluoranthene	202.25	110	375	72.5	28.7	162	168.4	132.6172
Acephenanthrylene	202.25	140	405.7	69.1	27.4	162.3	188.6	145.3825
Naphthacene	228.3	–	436.7	79.8	31.6	–	209.1	211.1320
Corannulene	250.3	–	438	93.5	37.1	170.6	210.1	180.8287
Pentacene	278.3	257	524.7	97.6	38.7	225.9	264.5	263.3969
Benzo[b]fluoranthene	252.3	166	467.5	90.3	35.8	196.1	228.6	214.6708
Benzo[k]fluoranthene	252.3	217	480	90.3	35.8	196.1	228.6	221.7297
Indeno[1,2,3-cd]pyrene	276.3	163.6	497.1	100.8	40	200.4	247.2	245.1506
Benzo[e]perelyne	252.3	177.5	467.5	90.3	35.8	196.1	228.6	202.6422
Anthanthrene	276.3	261	497.1	100.8	40	200.4	247.2	263.6018
Dibenz[a,c]anthracene	278.3	206	518	97.6	38.7	225.9	264.5	262.1866
Dibenz[a,j]anthracene	278.3	196	524.7	97.6	38.7	225.9	264.5	279.8474
Coronene	300.4	–	525.6	111.4	44.1	204.7	265.2	259.3191
Dibenzo[a,h]pyrene	302.4	308	552.3	108.1	42.9	230.2	282	300.3360
Dibenzo[a,i]pyrene	302.4	281.5	552.3	108.1	42.9	230.2	282	330.0352
Dibenzo[a,l]pyrene	302.4	162.4	552.3	108.1	42.9	230.2	282	267.1848
Rubicene	326.4	306	579	118.7	47	234.5	298.8	327.4316
Isochrysene	228.3	199	425	79.8	31.6	191.8	209.1	174.3036
5-Methylchrysene	242.3	118	449.4	84.6	33.5	208.1	217.8	206.2686

**Fig. 2:**
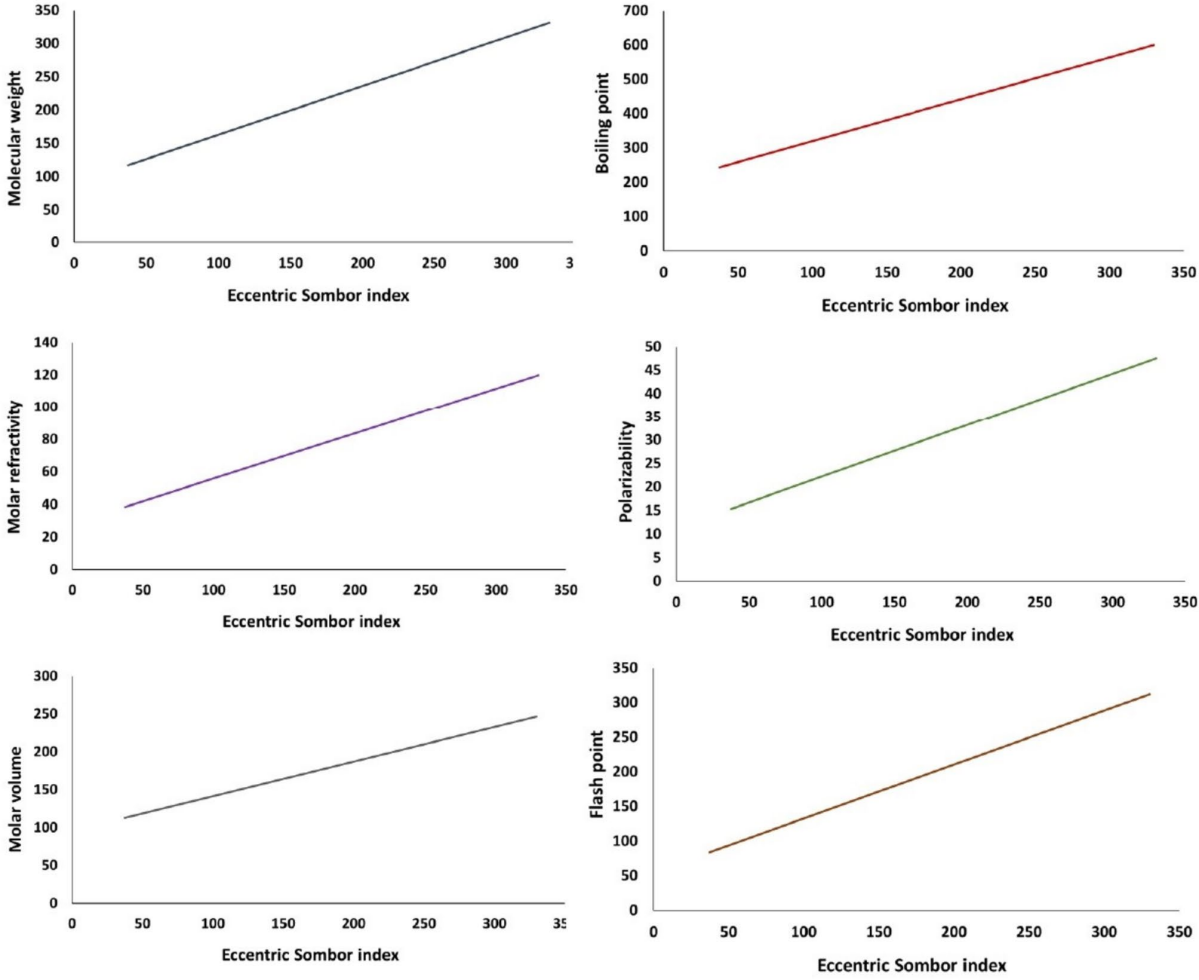
Linear relationship of eccentric Sombor index with various physicochemical properties.

The scatter plots for the above collection of data are given in Fig. 2. For each of the parameters, a linear regression model is derived, and the coefficient of determination $R^2$ is given.

## Comparisons and discussion

This study effectively predicted 32 of the most abundant polycyclic aromatic compounds using the eccentricity-based Sombor index. We have considered 7 physico-chemical properties of polycyclic aromatic compounds, among which, the molecular weight, polarizability, and flash point yield the highest coefficient of determination. Other properties like molar refractivity and molar volume also have a relatively strong correlation. But melting point and complexity have a weak correlation with eccentric Sombor index. The detailed list is given below.

**Table:** 

Properties	Regression equation	$R^2$
Molecular weight	$MW = 0.7307~SO^{\xi }(G)+90.099$	0.9571
Melting point	$MP = 0.8317 ~SO^{\xi }(G) + 12.087$	0.7282
Boiling point	$BP = 1.2171 ~SO^{\xi }(G) + 198.19$	0.9159
Molar refractivity	$MR= 0.2775 ~SO^{\xi }(G) + 28.229$	0.9370
Polarizability	$PO=0.1100~SO^{\xi }(G)+ 11.195$	0.9371
Molar volume	$MV=0.4575~SO^{\xi }(G)+ 95.528$	0.9422
Flash point	$FP=0.779~SO^{\xi }(G)+ 54.883$	0.9502

In^44^, the authors carried out a similar study and hence determined the impact of the degree-based Sombor index on the physico-chemical properties of polycyclic aromatic compounds. In comparison with the degree-based Sombor index, it was found that the eccentricity-based Sombor index yields higher predictive power. Clearly, we can see that molar volume yields a higher coefficient of determination ($R^2=0.9422$) than that of the degree-based Sombor index (0.7786). The comparison between the coefficient of determination values of degree-based and eccentricity-based Sombor index is given in the following Table 2.

**Table 2:** Comparison between the coefficient of determination values ($R^2$) of degree-based and eccentricity-based Sombor index.

Sombor index	MW	BP	MP	MR	PO	MV	FP
Degree-based	0.9713	0.7978	0.7009	0.9030	0.9029	0.7786	0.8595
Eccentricity-based	0.9571	0.9159	0.7282	0.9370	0.9371	0.9422	0.9502

## Conclusion

The output of the above analysis demonstrates that the eccentric Sombor index shows a fine and adequate linear relationship ($$R^2>0.9)$$ with most of the properties. The results obtained in this article indicate that the Sombor index achieves better correlation with physicochemical properties when vertex degrees are replaced by their corresponding eccentricities.

## Limitations and scope for future work

Although the QSPR study of eccentric Sombor index exhibits strong predictive ability for several physicochemical properties of polycyclic aromatic hydrocarbons, the present study has certain limitations.

- The analysis is restricted to polycyclic aromatic hydrocarbons, and therefore, the predictive capability of the eccentric Sombor index for other classes of chemical compounds remains to be explored.
- The regression analysis employed in this work is limited to linear models, although high coefficients of determination $R^2>0.9$ were obtained. Other regression analyses, such as logarithmic, exponential, quadratic, etc, can also be adopted.
- Since the dataset used for validation is finite, analysing a larger and more diverse set of compounds would enable a more reliable and applicable approach across different molecular classes.

Extending the study of the eccentric Sombor index to other classes of compounds beyond polycyclic aromatic hydrocarbons to evaluate its predictive power for a wider range of molecular structures would be a promising direction for future work. Conducting similar studies for several degree-based topological indices by replacing vertex degrees with their corresponding eccentricities may lead to improved correlations and, consequently, contribute more significantly to the QSPR analysis of chemical compounds.

## Data Availability

All data generated or analysed during this study are included in this article.
